# Cocaine Self-Administration Influences Central Nervous System Immune Responses in Male HIV-1 Transgenic Rats

**DOI:** 10.3390/cells11152405

**Published:** 2022-08-04

**Authors:** Chiomah Ezeomah, Chanida Fongsaran, Amanda L. Persons, T. Celeste Napier, Irma E. Cisneros

**Affiliations:** 1 Department of Pathology, University of Texas Medical Branch, 301 University Blvd, Galveston, TX 77550, USA; 2 Galveston National Laboratory, Institute of Human Infections and Immunity, University of Texas Medical Branch, 301 University Blvd, Galveston, TX 77550, USA; 3 Population Health Sciences, Weill Cornell Medical College of Cornell University, 402 East 67th Street, New York, NY 10065, USA; 4 NeuroInfectious Diseases, University of Texas Medical Branch, 301 University Blvd, Galveston, TX 77550, USA; 5 Physician Assistant Studies, Rush University Medical Center, Chicago, IL 60612, USA; 6 Center for Compulsive Behavior and Addiction, Rush University Medical Center, Chicago, IL 60612, USA; 7 Department of Psychiatry and Behavioral Sciences, Rush University Medical Center, Chicago, IL 60612, USA; 8 Center for Addiction Research, University of Texas Medical Branch, 301 University Blvd, Galveston, TX 77550, USA

**Keywords:** HIV-1, cocaine, neuroinflammation, central nervous system

## Abstract

Cocaine use increases the neurotoxic severity of human immunodeficiency virus-1 (**HIV-1**) infection and the development of HIV-associated neurocognitive disorders (**HAND**). Among the studied cellular mechanisms promoting neurotoxicity in HIV-1 and cocaine use, central nervous system (**CNS**) immunity, such as neuroimmune signaling and reduced antiviral activity, are risk determinants; however, concrete evidence remains elusive. In the present study, we tested the hypothesis that cocaine self-administration by transgenic HIV-1 (**HIV-1_Tg_**) rats promotes CNS inflammation. To test this hypothesis, we measured cytokine, chemokine, and growth factor protein levels in the frontal cortex (f**CTX**) and caudal striatum (c**STR**). Our results demonstrated that cocaine self-administration significantly increased fCTX inflammation in HIV-1_Tg_ rats, but not in the cSTR. Accordingly, we postulate that cocaine synergizes with HIV-1 proteins to increase neuroinflammation in a region-selective manner, including the fCTX. Given the fCTX role in cognition, this interaction may contribute to the hyperimmunity and reduced antiviral activity associated with cocaine-mediated enhancement of HAND.

## 1. Introduction

The development of human immunodeficiency virus (**HIV**)-associated neurocognitive disorders (**HAND**) remains a clinical burden among HIV-1 infected individuals [1]. Combined antiretroviral therapies (**cART**) do not completely mitigate the impact of HIV-1 on the central nervous system (**CNS**) [2]. There is a high prevalence of HIV-1 comorbidities, including drug addiction, that heighten the risk for HIV-1 infections and the acceleration of HIV/AIDS [3,4,5,6,7]. For example, cocaine can target immune cells such as macrophages and lymphocytes to impair the host immune response and enhance HIV viral replication [8,9,10]. Cocaine presumably enhances the development of HAND via increased neuroimmune signaling and CNS inflammation [11]; however, the combined impact of cocaine and HIV-1 on heightened immune responses remains unclear. 

Despite the control of HIV-1 replication during cART, chronic inflammation persists [12] and is associated with disease progression and neurocognitive disorders in people infected with HIV [13,14]. Levels of inflammatory cytokines including interleukin (**IL**)-6, IL-1β, tumor necrosis factor (**TNF**)-α and interferon (**IFN**)-γ remain elevated in HIV-1-infected patients receiving cART compared to age-/sex-matched controls [12,15]. Postmortem findings in the brain of HIV+ patients show astrocyte and microglia activation (hallmark characteristics of neuroinflammation) and elevated levels of IL-6, IL-1β, and TNF-α [16,17,18]. In vitro studies demonstrate that neurotoxic HIV-1 proteins, including Tat and gp120, can induce neuroinflammation to promote HAND-related neurotoxicity [19,20,21]. HIV-1 proteins enhance the activation of proinflammatory signal transduction pathways and translocation of nuclear factor kappa B (**NF-ĸB**) and TNF-α signaling, thereby triggering inflammatory signal transduction pathways [22,23,24].

Post-mortem brains collected from cocaine users have evidence of microglia and astrocyte activation [25]. Cocaine users also have increased circulating levels of IL-6 [26]. Cocaine activates NF-ĸB signaling and downstream increases of IL-1β, IL-6, IL-12, and TNF-α via interactions with toll-like receptors [27,28]. Cocaine self-administering rats demonstrate increased gene expression of inflammatory modulators in brain reward structures such as the prefrontal cortex (**pfCTX**) and striatum (**STR**) [29,30,31]. 

Although HIV-1 and cocaine individually modulate host systemic and CNS immune responses, the relationship between HIV-1 and cocaine in host CNS immunity remains unclear. Cocaine increases HIV-1 replication through dysregulation of innate and adaptive immunity, including the decline of CD4+ T cell counts [32,33,34,35]. Systemically, cocaine modulates levels of inflammatory cytokines and chemokines generated from peripheral blood mononuclear cells (**PBMC**), including: regulated upon activation T expressed and secreted (**RANTES**), macrophage inflammatory protein (**MIP**)-1α and MIP-1β [35] or via involvement of transforming growth factor beta (**TGF-β**) [8]. Other groups show that cocaine mitigates HIV-induced immunopathogenesis through downregulation of miRNA-155, thereby reducing IFN-γ production [36]. Clinical and preclinical in vivo neuroinflammatory studies are less widely available; however, in vitro studies demonstrate that cocaine enhances HIV-1 replication in brain resident immune cells, including astrocytes and microglia [37,38,39]. Proposed mechanisms of cocaine-induced increases of neuroinflammation include the generation of platelet monocyte complexes, cellular oxidative stress, excitotoxicity, and mitochondrial toxicity, which are all linked to activation of inflammatory signal transduction pathways and increased production of cytokines and chemokines [40,41,42,43]. Therefore, cocaine may act synergistically with HIV-1 proteins to enhance neuroinflammation. However, there remains ambiguity in the impact of cocaine on HIV-1 related neuroinflammation. 

It is critical to understand the neuroimmune comorbidity between HIV-1 and cocaine to provide novel avenues for exploring therapeutics to combat the hyperactive immune response. Therefore, we utilized a well-established rodent model of HIV-infected humans, the HIV-1 transgenic (**HIV-1_Tg_**) rat. We tested the hypothesis that cocaine self-administration exacerbates frontal cortex (**fCTX**)- and caudal striatum (**cSTR**)-related inflammation in HIV-1_Tg_ rats. The fCTX and cSTR were evaluated as HIV-1-induced neuropathological features in these brain regions are related to neurological decline in HIV-1 patients [44,45,46,47,48]. HIV-1_Tg_ rats are well suited for this study in part because HIV-1 protein and mRNA (Tat, gp120, Nef and Vif) occur in the fCTX [49]. Self-administration by rats models aspects of human drug-taking, including self-initiation and self-titration, which demonstrate the desire and willingness to take drugs. The HIV-1_Tg_ rats can be trained to self-administer cocaine to model cocaine use in HIV-infected humans [50,51]. Here we used male HIV-1_Tg_ or non-transgenic wild-type (**WT**) Fischer 344 rats (**F344**) that self-administered cocaine or were saline-yoked. We measured cytokines levels, described to be elevated during HIV-1 infection in humans [12,13], in the fCTX and cSTR. Results from this study provide critical information on immunomodulatory effects of cocaine and its impact on HIV-1-mediated neuropathogenesis. 

## 2. Materials and Methods

### 2.1. Self-Administration

Rat brain tissues, harvested from male HIV-1_Tg_ (*n = 15*) and (WT) F344 (*n = 12*) rats, were obtained from a specimen repository in the laboratory of Dr. T. Celeste Napier (Rush University Medical Center, Chicago IL). Surgical and self-administration procedures generally followed previously published protocols [51,52]. In brief, rats were purchased from Envigo Laboratories (Indianapolis, IN, USA) and housed in genotype- and treatment-similar pairs; food and water were provided ad libitum. Rats were implanted with custom-made silastic catheters (0.3 mm ID × 0.64 mm OD; Dow Corning Co., Midland, MI, USA) inserted into the right jugular vein. The distal end of the catheters extended subcutaneously over the mid-scapular region and exited through a metal guide cannula (22 gauge; Plastics One Inc., Roanoke, VA, USA) anchored to a subcutaneously implanted vinyl mesh. The duration of post-surgery recovery lasted at least 7 days, during which catheters were flushed daily with 0.1–0.2 mL sterile saline to maintain patency. Self-administration took place in ventilated, sound-attenuating operant chambers equipped with two ‘nose-poke’ holes, a stimulus light above each hole, an audio tone generator, and a house light (Med-Associates, St. Albans, VT, USA). Operant sessions were conducted 2 h/day for a total of 14 days on a fixed-ratio 1 (FR1) schedule of reinforcement. A nose-poke in the active hole resulted in a 6 sec infusion of cocaine (1.0 mg/kg/0.1 mL), delivery of an audio tone, and illumination of the stimulus light. Nose-pokes in the inactive hole had no programmed consequence. Control rats were yoked to a cocaine counterpart of the same genotype, receiving a non-contingent infusion of saline (1.0 mL) each time their counterpart self-administered cocaine. For saline-yoked rats, nose-pokes in either hole were recorded but had no programmed consequence. Brain tissues were harvested one day after concluding the operant task, fast-frozen on dry ice, and stored at −80 °C. Rats were handled in accordance with the procedures established in the Guide for the Care and Use of Laboratory Animals (National Research Council, Washington DC, USA) as approved by the Rush University Institutional Animal Care and Use Committee.

### 2.2. Dataset

A total of 27 rats were used for this study. From each rat, fCTX and caudal STR were isolated. Cytokine expressions were measured from brain regions that included the fCTX and cSTR of 15 HIV-1_Tg_ rats and 12 or 9 WT rats, respectively. Multiplex immunoassay results that were out of range (not within the linear range of the standard curve) were removed from the dataset prior to analysis.

### 2.3. Protein Extraction

fCTX and cSTR brain tissues were homogenized and aliquoted for protein using the Qiagen Tissuelyser for 30 s in 200–500 µL ice-cold homogenization/extraction buffer, volume specific to brain region (20 mM HEPES, 200 mM NaCl, 1 mM EDTA, 1 mM DTT, 10 µL/mL phosphatase inhibitor cocktail 2 (Sigma, Cat #P5726, Burlington, MA, USA), 10 µL/mL phosphatase inhibitor cocktail 3 (Sigma, Cat #P0044), RNase inhibitor [53]). Homogenized protein aliquots were centrifuged at >8000 rpm for 15 min. The pellet was resuspended in mammalian protein extraction reagent (ThermoFisher Scientific, Waltham, MA, USA), supplemented with protease inhibitor, 10 µL/mL phosphatase inhibitor cocktail 2 (Sigma, Cat #P5726), 10 µL/mL phosphatase inhibitor cocktail 3 (Sigma, Cat #P0044), RNase inhibitor) and resuspended by manual pipetting. Protein concentrations were quantified using Precision Red Advanced Protein Assay (Cytoskeleton, Inc. Denver, CO, USA) per manufacturers’ instructions. 

### 2.4. Quantification of Brain Innate Immune Proteins by BioPlex

Cytokine levels in rat brain protein lysate were measured using the Bio-Plex Pro Rat Cytokine Group I Panel 23-plex assay (BioRad, Cat # 12005641, Philadelphia, PA, USA) following the manufacturer’s instructions and as previously described [53]. Briefly, rat brain lysates were diluted 1:4 in Bio-Plex sample diluent (containing BSA to a final concentration of 0.5%). Standards were reconstituted and coupled beads were prepared following manufacturer’s instructions. Approximately 50 µL of coupled beads was added to each well in a 96-well plate, then washed prior to adding 50 µL of standard and samples (both assayed in duplicate) to the appropriate well in a 96-well plate. The plate was incubated and washed following manufacturers’ instructions, then read using a Bio-Plex 200 system. Innate immune protein concentrations were normalized to total protein as measured by Precision Red Advanced Protein Assay (Cytoskeleton, Inc.). Assay sensitivity and limit of detection (pg/mL) for each target: G-CSF (0.2), VEGF (0.3), IL-7/M-CSF (0.4), GM-CSF/GRO-KC (0.6), MIP-1α (0.7), IL-13 (0.9), IFN-γ/IL-1α/IL-4/ (1.0), IL-1β (2.0), IL-2/RANTES/TNF-α (3.0), IL-18/MCP-1 (4.0), IL-10 (5.0), IL-5 (6.0), IL-6 (10.0), and MIP-1α (12.0). 

### 2.5. Statistical Analyses

Cumulative cocaine intake was compared between groups using a two-sample *t*-test. Protein expression levels measured in tissue lysates were not normally distributed; we therefore performed a log_2_ transformation for all variables prior to performing *t*-tests and linear regression analysis. In addition, prior to performing t-tests, the data were stratified by rat model. Two-sample *t*-tests with unequal variance were used to compare the difference in mean expression levels of each cytokine in HIV-1_Tg_ rats that self-administered cocaine versus saline-yoked rats or WT rats with cocaine self-administration versus saline-yoked rats. Bonferroni correction was used for multiple testing correction of *p*-values obtained from the t-tests. The expression levels of each cytokine/chemokine, stratified by rat model (WT vs. HIV-1_Tg_), were described using mean and standard deviation. Additionally, we performed linear regression analysis to evaluate main and interaction effects of treatment–cocaine versus saline (reference) and genotype, HIV-1_Tg_ versus WT (reference), on cytokine and chemokine expression levels. All tests were conducted in R (version 3.6.1) and an alpha level of <0.05 defined statistical significance and were two-tailed.

## 3. Results

### 3.1. Cocaine Self-Administration in WT and HIV-1_Tg_ Rats

The average cumulative cocaine intake in the WT rats and HIV-1_Tg_ rats was 112.2 ± 13.6 mg/kg and 121.3 ± 13.6 mg/kg, respectively. There was no difference between genotypes with respect to cocaine intake (*p* = 0.65), which is consistent with our prior reports on cocaine [51] and methamphetamine [54] self-administration in these rats.

### 3.2. Cocaine Self-Administration Increases Pro- and Anti-Inflammatory Cytokine Generation in the fCTX of HIV-1_Tg_ Rats

**Figure 1:**
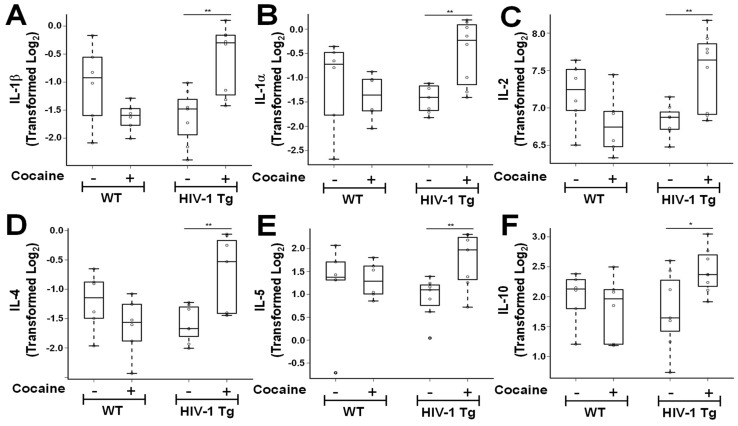
Cocaine self-administration regulates cortical interleukin levels in HIV-1_Tg_ rats. Equivalent total protein lysates from the frontal cortex (fCTX) were assayed for interleukin levels from WT saline-yoked (*n =* 7), WT cocaine SA (*n =* 8), HIV-1_Tg_ saline-yoked (*n =* 6), and HIV-1_Tg_ cocaine SA (*n* = 6) male rats. Protein levels for IL-1β (**A**), IL-1α (**B**), IL-2 (**C**), IL-4 (**D**), IL-5 (**E**), and IL-10 (**F**) are shown. Log_2_ transformation was performed prior to statistical analyses since some expression levels were not normally distributed. * p < 0.05, ** p < 0.01.

**Figure 2:**
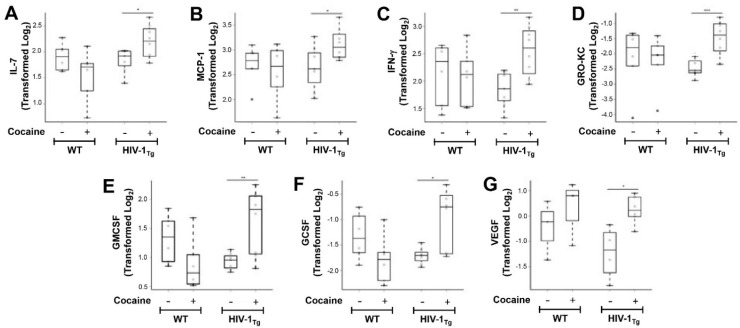
Cocaine self-administration regulates cortical interleukin and growth factor levels in HIV-1_Tg_ rats. Frontal cortex (fCTX) brain tissue was collected, and equivalent total protein lysates were assayed for innate immune markers from WT saline-yoked (*n =* 7), WT cocaine SA (*n =* 8), HIV-1_Tg_ saline-yoked (*n =* 6), and HIV-1_Tg_ cocaine SA (*n =* 6) male rats. Protein levels for IL-7 (**A**), MCP-1 (**B**), IFN-γ (**C**), GRO-KC (**D**), GM-CSF (**E**), G-CSF (**F**), and VEGF (**G**) are shown. Log_2_ transformation was performed prior to statistical analyses since some expression levels were not normally distributed. * p < 0.05, ** p < 0.01, *** p < 0.001.

Multiplex data demonstrated that total protein assayed from fCTX showed expression of several cytokines (pro- and anti-inflammatory interleukins, chemokines, and growth factors). Comparisons between HIV-1_Tg_ rats that self-administered cocaine to saline-yoked HIV-1_Tg_ rats demonstrated significantly higher protein levels of IL-1β (Figure 1A, ** p < 0.01), IL-1α (Figure 1B, ** p < 0.01), IL-2 (Figure 1C, ** p < 0.01), IL-4 (Figure 1D, ** p < 0.01), IL-5 (Figure 1E, ** p < 0.01), IL-10 (Figure 1F, * p < 0.05), IL-7 (Figure 2A, * p < 0.05), MCP-1 (Figure 2B, * p < 0.05), IFN-γ (Figure 2C, ** p < 0.01), GRO/KC (Figure 2D, *** p < 0.001), GM-CSF (Figure 2E, ** p < 0.01), G-CSF (Figure 2F, * p < 0.05), and VEGF (Figure 2G, * p < 0.05). No significant changes were obtained for IL-6 (Supplementary Figure S1A), IL-12 (Supplementary Figure S1B), IL-18 (Supplementary Figure S1C), TNF-α (Supplementary Figure S1D), M-CSF (Supplementary Figure S1E), or MIP-3α (Supplementary Figure S1F).

Among the 23 cytokines assayed, IFN-γ, IL-1α, IL-1β, IL-2, IL-4, IL-5, IL-6, IL-7, IL-10, IL-12, G-CSF, GM-CSF, GRO/KC, M-CSF, MCP-1, MIP3α, RANTES, TNFα, VEGF and IL-18 were expressed in the STR of WT male rats that were saline-yoked or that self-administered cocaine, but no differences were obtained between genotype or treatment groups (Supplementary Table S1). 

In HIV-1_Tg_ rats, cocaine elevated expression levels of G-CSF (*p =* 0.011); GM-CSF (*p =* 0.010); GRO/KC (*p =* 0.001); IFN-γ (*p =* 0.004); IL-1β (*p =* 0.004); IL-1α (*p =* 0.003); IL-2 (*p =* 0.010); IL-4 (*p =* 0.005); IL-5 (*p =* 0.008); IL-7 (*p =* 0.023); and IL-10 (*p =* 0.044); MCP-1 (*p =* 0.043); and VEGF (*p =* 0.038) (Table 1). By contrast, no statistically significant differences were detected in the expression of IL-6, IL-12 or IL-18 following cocaine self-administration compared with saline-yoked controls in HIV-1_Tg_ rats. Among WT rats, no statistically significant difference in mean expression levels of any cytokine/chemokine was observed between saline-yoked and cocaine self-administration.

Interaction effects were observed. Specifically, we adjusted for HIV-1 protein, and compared HIV-1_Tg_ saline with HIV-1Tg cocaine, given that we found neither WT cocaine nor HIV-1_Tg_ saline (after adjustment) to independently result in statistically significant differences in cytokine expression when compared to WT saline. We observed that cocaine synergized with HIV-1 to increase expression (Table 2) of G-CSF (interaction effect: *p =* 0.003), GM-CSF (interaction effect: *p =* 0.002), IFN-γ (interaction effect: *p =* 0.042), IL-1α (interaction effect: *p =* 0.017), IL-1β (interaction effect: *p =* 0.001), IL-2 (interaction effect: *p =* 0.003), IL-4 (interaction effect: *p =* 0.003), IL-7 (interaction effect: *p =* 0.010), IL-10 (interaction effect: *p* = 0.046), MIP-3α (interaction effect: *p =* 0.021), and TNF-α (interaction effect: *p =* 0.019). Neither WT cocaine nor HIV-1_Tg_ saline (after adjustment) independently resulted in statistically significant differences in expression of each of these cytokines when compared to WT saline.

## 4. Discussion

The contribution of inflammation in the CNS likely plays a significant role in the development of HAND, which is exacerbated during drug addiction [3,4]. Findings from clinical studies are not always consistent likely reflecting the heterogeneity of patient populations and differences in use history. To help control these factors, we used an HIV−1 rodent model and implemented operant self−administration protocols to elucidate the impact of cocaine self−administration on HIV−1 related neuroinflammation. The HIV−1_Tg_ rat is a noninfectious rodent model of HIV infection that allows for chronic lifelong exposure to viral proteins including Tat and gp120 and develops characteristic immune deficiencies [52,55,56,57]. This may pose some limitations to this model, given that it does not recapitulate cART−induced suppression of viral gene expression as seen with treated HIV−infected patients. We demonstrated that cocaine−self−administration negatively impacted particular factors for HIV−1 related CNS inflammation, within brain regions that are involved in the development of HAND. These outcomes point to mechanisms of hyperinflammation and reduced antiviral activity that may occur during HIV−1 infection and cocaine comorbidity.

In the absence of cocaine, we did not find any differences in inflammatory markers between the WT and HIV−1_Tg_ groups. This was an unexpected outcome, as lifelong exposure to viral proteins in HIV−1_Tg_ rats are associated with neurological and behavioral deficits that are characteristic of human HIV−1 infections, and because viral proteins can activate host immune responses [58,59]. Repunte−Canonigo et al. reported increased expression of astrocyte and microglia activation markers, GFAP and Iba1, in the brains of HIV−1_Tg_ rats compared to WT controls, which indicates activation of neuroinflammatory processes [60]. However, they did not find pro−inflammatory genes to be differentially expressed except for the antiviral gene, interferon stimulated gene (**ISG**)15 [60]. Gene expression levels of the chemokine monocyte chemoattract protein−1 (*Mcp−1/Ccl2*) are significantly increased in the hippocampus of HIV−1_Tg_ rats compared to WT, but no significant differences in gene expression levels of *Il−1β, NF−ĸB* or *Tnf* were detected [61]. Reid et al. showed an age−dependent loss in reactive phenotypes of microglia and astrocytes in the STR and corpus callosum of HIV−1_Tg_ compared to WT controls [62], but these authors did not measure inflammatory responses. Our findings are also consistent with reports demonstrating no differences in cytokine/chemokine levels measured for STR and hippocampal tissue lysates of 3−month−old and 9−month−old male WT and HIV−1_Tg_ rats [63]. Thus, changes in cytokine levels in the brains of WT and/or HIV−1_Tg_ rats following cocaine self−administration strongly implicate neuroimmune activation following cocaine exposure. 

In humans, cocaine increases the onset and severity of HAND (reviewed in [64]). This likely reflects the impact of cocaine on CNS inflammation, given that chronic low levels of neuroinflammation exacerbate neurotoxicity [65,66]. Levels of pro−inflammatory cytokines, IL−1β, TNF−α and IL−6, are elevated in humans with cocaine use disorder [26,67,68,69]. In rats non−contingently exposed to cocaine (i.p.), elevated levels of IL−1β are measured in the pfCTX and nucleus accumbens [27]. In rats that self−administered cocaine, IL−1β and TNFα are increased in the ventral tegmental area (VTA) [70]. Another study demonstrated that inflammatory markers, including IL−1β, IL−1α, IL−2, IL−4, IL−6, IL−10, IL−12, and IFNγ, are enhanced in the pfCTX, STR, and VTA of rats that self−administered cocaine, but only in the presence of traumatic brain injury [71]. Although we did not observe an effect of cocaine on fCTX or STR neuroinflammatory mediators in WT rats, we found that cocaine−self−administration resulted in a hyperimmune response in the fCTX of HIV−1_Tg_ rats, with higher levels occurring in 13 cytokines, chemokines, and growth factors. Our results indicate an interactive effect of HIV−1 toxic proteins (e.g., Tat, gp120) and cocaine on neuroinflammation. There are limited clinical and preclinical studies demonstrating the impact of cocaine and HIV−1 on the neuroimmune response; however, there is evidence that drug dependency enhances HIV−1 related cognitive impairments [72], which is negatively impacted by inflammation [73]. 

Taken together, our results demonstrate that in this HIV−1_Tg_ rat model of cocaine use disorder, HIV−1 proteins or cocaine alone are not enough to initiate a neuroinflammatory response in the fCTX or cSTR. However, in the fCTX, the hyper−neuroimmune response during combined exposure of cocaine and HIV−1 indicates that rather than an additive effect of cocaine on HIV−1 related neuroinflammation, there is a synergistic relationship between cocaine and HIV−1 on the neuroimmune response. 

## Figures and Tables

**Table 1:** **Frontal cortex (fCTX) cytokine level summary statistics stratified by rat model WT vs. HIV-1_Tg_.** Student’s *t*-tests with unequal variance were used to compare the difference in mean expression levels (and SD) of each cytokine, chemokine, or growth factor in WT (saline-yoked (*n =* 7) and cocaine SA (*n =* 8)) and HIV-1_Tg_ (saline-yoked (*n* = 6) and cocaine SA (*n* = 6)) in the fCTX. The 95% CI, overall *p* value and cumulative replicates are given in the table. Bold cytokines, chemokines, or growth factors indicate targets that were significantly increased in HIV-1_Tg_ rats compared to WT.

	WT					HIV-1_Tg_				
Cytokine (log_2_ Transformed)	Saline	Cocaine	95% CI	*p*-Value	Adjusted *p*-Value	Saline	Cocaine	95% CI	*p*-Value	Adjusted *p*-Value
	Mean (SD)	Mean (SD)			(Bonferroni)	Mean (SD)	Mean (SD)			(Bonferroni)
**G−CSF**	−1.33 (0.45)	−1.79 (0.47)	[0.218, 1.23]	0.114	0.110	−1.71 (0.16)	−0.99 (0.60)	[−1.04, 0.131]	**0.011**	**0.011**
**GM−CSF**	1.32 (0.40)	0.88 (0.44)	[0.216, 1.55]	0.097	0.097	0.93 (0.14)	1.62 (0.56)	[−1.80, 0.097]	**0.01**	**0.010**
**GRO/KC**	−2.14 (1.06)	−2.24 (0.86)	[0.505, 1.48]	0.852	0.850	−2.46 (0.29)	−1.46 (0.55)	[−1.35, 1.14]	**0.001**	**0.001**
**IFN−γ**	2.15 (0.56)	2.08 (0.51)	[0.266, 1.15]	0.828	0.830	1.85 (0.33)	2.55 (0.46)	[−0.758, 0.620]	**0.004**	**0.040**
**IL−1α**	−1.12 (0.91)	−1.39 (0.47)	[0.386, 1.53]	0.537	0.540	−1.43 (0.29)	−0.47 (0.66)	[−1.23, 0.707]	**0.004**	**0.004**
**IL−1β**	−1.04 (0.70)	−1.62 (0.25)	[0.421, 1.65]	0.102	0.100	−1.62 (0.50)	−0.59 (0.60)	[−1.31, 0.155]	**0.003**	**0.003**
**IL−2**	7.19 (0.42)	6.78 (0.41)	[0.194, 1.10]	0.123	0.120	6.83 (0.22)	7.48 (0.52)	[−0.933, 0.130]	**0.01**	**0.010**
**IL−4**	−1.21 (0.49)	−1.63 (0.48)	[0.327, 1.42]	0.169	0.170	−1.59 (0.32)	−0.72 (0.61)	[−1.04, 0.209]	**0.005**	**0.005**
**IL−5**	1.19 (0.98)	1.31 (0.39)	[0.259, 1.42]	0.784	0.780	0.92 (0.46)	1.76 (0.58)	[−0.906, 1.15]	**0.008**	**0.008**
IL−6	0.84 (0.88)	0.87 (0.65)	[−0.275, 1.30]	0.95	0.950	0.71 (0.77)	1.22 (0.59)	[−0.978, 1.04]	0.18	0.180
**IL−7**	1.90 (0.26)	1.55 (0.49)	[0.058, 0.673]	0.155	0.150	1.83 (0.23)	2.20 (0.31)	[−0.879, 0.168]	**0.023**	**0.023**
**IL−10**	1.99 (0.43)	1.82 (0.53)	[0.024, 1.30]	0.565	0.560	1.77 (0.66)	2.43 (0.37)	[−0.790, 0.458]	**0.044**	**0.044**
IL−12	0.14 (0.77)	0.05 (0.88)	[−0.117, 1.40]	0.857	0.860	0.16 (0.80)	0.81 (0.40)	[−1.16, 0.978]	0.088	0.088
IL−18	2.57 (0.08)	2.65 (0.38)	[−0.671, 1.31]	0.711	0.710	2.44 (0.81)	2.76 (0.40)	[−0.510, 0.667]	0.449	0.450
M−CSF	−2.97 (0.35)	−3.15 (0.39)	[−0.261, 0.418]	0.425	0.430	−2.98 (0.34)	−2.90 (0.24)	[−0.656, 0.300]	0.618	0.620
**MCP−1**	2.70 (0.39)	2.56 (0.56)	[0.018, 0.927]	0.608	0.610	2.64 (0.46)	3.11 (0.31)	[−0.781, 0.484]	**0.043**	**0.043**
MIP−3α	−1.28 (0.27)	−1.81 (0.52)	[−0.188, 0.614]	0.059	0.059	−1.24 (0.34)	−1.02 (0.37)	[−1.08, 0.026]	0.272	0.270
TNF−α	5.23 (0.16)	4.95 (0.39)	[−0.032, 0.477]	0.137	0.140	5.19 (0.18)	5.41 (0.27)	[−0.697, 0.120]	0.081	0.081
**VEGF**	−0.46 (1.17)	0.41 (1.08)	[0.144, 3.24]	0.366	0.370	−1.45 (1.04)	0.24 (0.56)	[−1.48, 3.24]	**0.038**	**0.038**

Footnote: For WT rats, each variable had 12 observations except IL−18 (8 observations) and VEGF (7 observations). For HIV−1_Tg_, each variable had 15 observations except IL−18 (13 observations) and VEGF (10 observations).

**Table 2:** Linear regression results for frontal cortex (fCTX) cytokine, chemokine, or growth factor levels with interaction effect. Linear regression analysis was performed to evaluate main and interaction effects of treatment–cocaine versus saline (reference) and genotype, HIV−1_Tg_ versus WT (referent) on cytokine, chemokine, or growth factor levels in the fCTX. Bold type indicates targets with significant interactions.

Variable (log_2_ Transformed)	HIV-1_Tg_ Cocaine Interaction (WT: Saline Referent) Estimate	*p*-Value	95% CI
**G−CSF**	**1.18**	**0.003**	**[0.45, 1.91]**
**GM−CSF**	**1.14**	**0.002**	**[0.46, 1.81]**
GRO/KC	1.1	0.06	[−0.05, 2.25]
**IFN−γ**	**0.78**	**0.042**	**[0.03, 1.52]**
**IL−1α**	**1.23**	**0.017**	**[0.24, 2.22]**
**IL−1β**	**1.62**	**0.001**	**[0.74, 2.49]**
**IL−2**	**1.05**	**0.003**	**[0.39, 1.71]**
**IL−4**	**1.29**	**0.003**	**[0.50, 2.08]**
IL−5	0.72	0.155	[−0.29, 1.78]
IL−6	0.48	0.397	[−0.67, 1.64]
**IL−7**	**0.72**	**0.01**	**[0.19, 1.25]**
**IL−10**	**0.83**	**0.046**	**[0.01, 1.64]**
IL−12	0.73	0.201	[−0.42, 1.88]
IL−18	0.24	0.6	[−0.71, 1.19]
M−CSF	0.26	0.322	[−0.27, 0.78]
MCP−1	0.62	0.076	[−0.07, 1.31]
**MIP−3α**	**0.74**	**0.021**	**[0.12, 1.36]**
**TNF−α**	**0.51**	**0.019**	**[0.09, 0.93]**
VEGF	0.81	0.393	[−1.17, 2.80]

Footnote: Each variable had 27 observations except IL−18 (21 observations) and VEGF (17 observations).

## Data Availability

All relevant data are within the paper and its supporting information.

## Supplementary Materials

The following supporting information can be downloaded at: https://www.mdpi.com/article/10.3390/cells11152405/s1, Figure S1: Cytokines with no significant difference in the fCTX of WT and HIV−1_Tg_ rats; Table S1. Caudal striatum (cSTR) cytokine level summary statistics stratified by rat model WT vs. HIV−1_Tg_.
