# Supplementary material for: Cocaine Self-Administration Influences Central Nervous System Immune Responses in Male HIV-1 Transgenic Rats
Source: Cells. 2022 Aug 4;11(15):2405. doi: 10.3390/cells11152405 (PMC9368446; doi:10.3390/cells11152405)
Supplement: Supplementary file 1 [file cells-11-02405-s001.zip › cells-1722726-supplementary-final-1.pdf]

Table S1. Caudal striatum (cSTR) cytokine level summary statistics stratified by rat model WT vs. HIV−1_{Tg}. Student's t−tests with unequal variance were used to compare the difference in mean expression levels (and SD) of each cytokine, chemokine, or growth factor in WT (saline−yoked (*n = 7*) and cocaine SA (*n = 8*)) and HIV−1_{Tg} (saline−yoked (*n = 6*) and cocaine SA (*n = 6*)) in the cSTR. The 95% CI, overall p value and cumulative replicates are given in the table. Bold cytokines, chemokines, or growth factors indicate targets that were significantly increased in HIV−1_{Tg} rats compared to WT.

	WT					HIV-1$_{Tg}$				
Cytokine (log$_2$ Transformed)	Saline Mean (SD)	Cocaine Mean (SD)	95% CI	p-value	Adjusted p-value (Bonferroni)	Saline Mean (SD)	Cocaine Mean (SD)	95% CI	p-value	Adjusted p-value (Bonferroni)
G−CSF	0.44 (0.81)	0.09 (0.63)	[−0.595, 1.29]	0.428	0.430	0.34 (0.70)	0.46 (0.45)	[−0.811, 0.574]	0.711	0.710
GM−CSF	3.03 (0.64)	2.70 (0.60)	[−0.46, 1.13]	0.369	0.370	2.44 (0.55)	2.59 (0.35)	[−0.610, 0.392]	0.555	0.560
GRO/KC	−0.49 (0.93)	−1.47 (1.14)	[−0.433, 2.40]	0.151	0.150	−2.56 (2.42)	−0.91 (0.68)	[−3.90, 0.602]	0.126	0.130
IFN−γ	3.60 (0.79)	3.23 (0.62)	[−0.543, 1.29]	0.383	0.380	3.71 (0.79)	3.91 (0.38)	[−0.948, 0.544]	0.551	0.550
IL−1α	−0.29 (1.35)	−0.38 (0.56)	[−1.34, 1.51]	0.894	0.890	−1.09 (1.17)	−0.79 (1.38)	[−1.79, 1.19]	0.666	0.670
IL−1β	0.01 (0.50)	−0.29 (0.77)	[−0.554, 1.16]	0.443	0.440	−0.06 (1.06)	0.28 (0.52)	[−1.35, 0.669]	0.463	0.460
IL−2	9.08 (0.41)	8.74 (0.48)	[−0.234, 0.92]	0.213	0.210	8.74 (0.34)	8.77 (0.19)	[−0.356, 0.297]	0.845	0.840
IL−4	−0.20 (0.47)	−0.57 (0.36)	[−0.181, 0.914]	0.165	0.160	−1.51 (2.62)	−0.471 (0.48)	[−4.28, 2.20]	0.428	0.430
IL−5	2.31 (0.89)	2.09 (0.75)	[−0.840, 1.29]	0.646	0.650	2.21 (0.58)	2.66 (0.22)	[−1.00, 0.09]	0.088	0.088
IL−6	2.05 (0.87)	1.13 (2.06)	[−1.27, 3.09]	0.352	0.350	1.67 (0.14)	0.694 (1.06)	[−0.676, 2.62]	0.162	0.160
IL−7	3.80 (0.70)	3.34 (0.64)	[−0.408, 1.33]	0.265	0.270	3.59 (0.65)	3.68 (0.37)	[−0.717, 0.530]	0.742	0.740
IL−10	3.56 (0.99)	3.15 (0.58)	[−0.657, 1.49]	0.396	0.400	4.98 (0.94)	5.25 (0.56)	[−1.18, 0.642]	0.523	0.520
IL−12	1.99 (1.12)	1.48 (1.10)	[−0.916, 1.94]	0.442	0.440	2.58 (0.80)	3.03 (0.52)	[−1.24, 0.338]	0.232	0.230
IL−18	4.06 (0.90)	3.77 (0.64)	[−0.762, 1.34]	0.548	0.550	3.44 (0.77)	3.67 (0.22)	[−0.947, 0.485]	0.469	0.470
M−CSF	−1.65 (0.84)	−1.82 (1.05)	[−1.06, 1.41]	0.756	0.760	−7.65 (3.45)	−5.42 (3.00)	[−7.57, 3.11]	0.347	0.350
MCP−1	4.17 (0.76)	3.73 (0.70)	[−0.491, 1.38]	0.313	0.310	3.44 (1.10)	3.90 (0.50)	[−1.49, 0.578]	0.339	0.340
MIP−3α	0.52 (0.72)	0.13 (0.77)	[−0.565, 1.36]	0.38	0.380	−0.75 (0.90)	−0.51 (0.57)	[−1.12, 0.636]	0.55	0.550
RANTES	−1.90 (0.65)	−3.27 (1.03)	[−3.37, 6.10]	0.272	0.270	−1.86 (1.09)	−1.47 (0.46)	[−2.04, 1.26]	0.535	0.540
TNF−α	6.76 (0.57)	6.46 (0.59)	[−0.453, 1.05]	0.396	0.400	6.15 (0.48)	6.18 (0.43)	[−0.549, 0.490]	0.904	0.900
VEGF	1.18 (3.94)	2.32 (3.54)	[−13.3, 11.0]	0.763	0.760	2.99 (1.40)	4.30 (0.87)	[−2.83, 0.199]	0.08	0.080

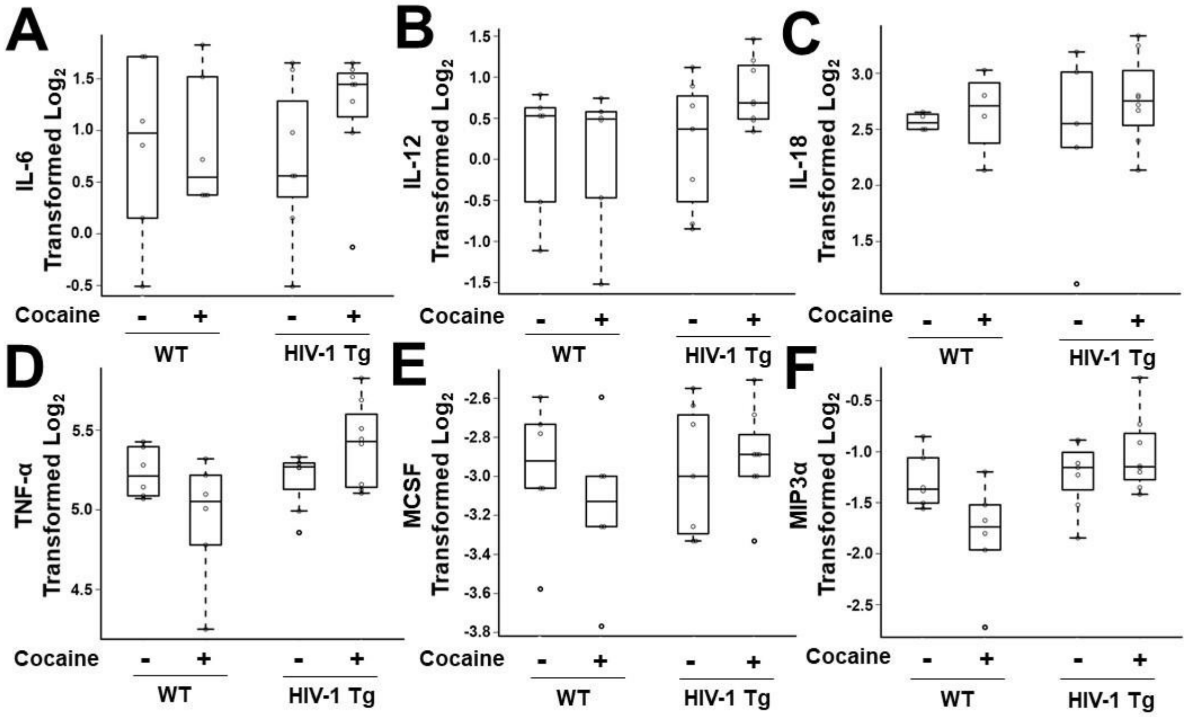

Figure S1. Cytokines with no significant difference in the fCTX of WT and HIV−1Tg rats. FCTX brain tissue was collected, and equivalent total protein lysates were assayed for cytokine levels from F344 saline-yoked (*n=6*), F344 cocaine SA (*n=6*), HIV-1Tg saline-yoked (*n=7*), and HIV-1Tg cocaine SA (*n=8*) male rats. Protein levels for IL-6 (**A**), IL-12 (**B**), IL-18 (**C**), TNF- (**D**), MCSF (**E**), and MIP3 (**F**) are shown. Log2 transformation was performed prior to statistical analyses since some expression levels were not normally distributed. All tests were conducted in R (version 3.6.1).
